# Photosynthate Regulation of the Root System Architecture Mediated by the Heterotrimeric G Protein Complex in *Arabidopsis*

**DOI:** 10.3389/fpls.2016.01255

**Published:** 2016-08-25

**Authors:** Yashwanti Mudgil, Abhijit Karve, Paulo J. P. L. Teixeira, Kun Jiang, Meral Tunc-Ozdemir, Alan M. Jones

**Affiliations:** ^1^Department of Botany, University of DelhiDelhi, India; ^2^Department of Biology, University of North Carolina at Chapel Hill, Chapel HillNC, USA; ^3^Brookhaven National Lab, UptonNY, USA; ^4^Department of Pharmacology, University of North Carolina at Chapel Hill, Chapel HillNC, USA

**Keywords:** photosynthetic partitioning, positron electron tomography imaging, AGB1, lateral root density, glucose, gene expression, PIN2-GFP

## Abstract

Assimilate partitioning to the root system is a desirable developmental trait to control but little is known of the signaling pathway underlying partitioning. A null mutation in the gene encoding the Gβ subunit of the heterotrimeric G protein complex, a nexus for a variety of signaling pathways, confers altered sugar partitioning in roots. While fixed carbon rapidly reached the roots of wild type and *agb1-2* mutant seedlings, *agb1* roots had more of this fixed carbon in the form of glucose, fructose, and sucrose which manifested as a higher lateral root density. Upon glucose treatment, the *agb1-2* mutant had abnormal gene expression in the root tip validated by transcriptome analysis. In addition, PIN2 membrane localization was altered in the *agb1-2* mutant. The heterotrimeric G protein complex integrates photosynthesis-derived sugar signaling incorporating both membrane-and transcriptional-based mechanisms. The time constants for these signaling mechanisms are in the same range as photosynthate delivery to the root, raising the possibility that root cells are able to use changes in carbon fixation in real time to adjust growth behavior.

## Introduction

**FIGURE 1:**
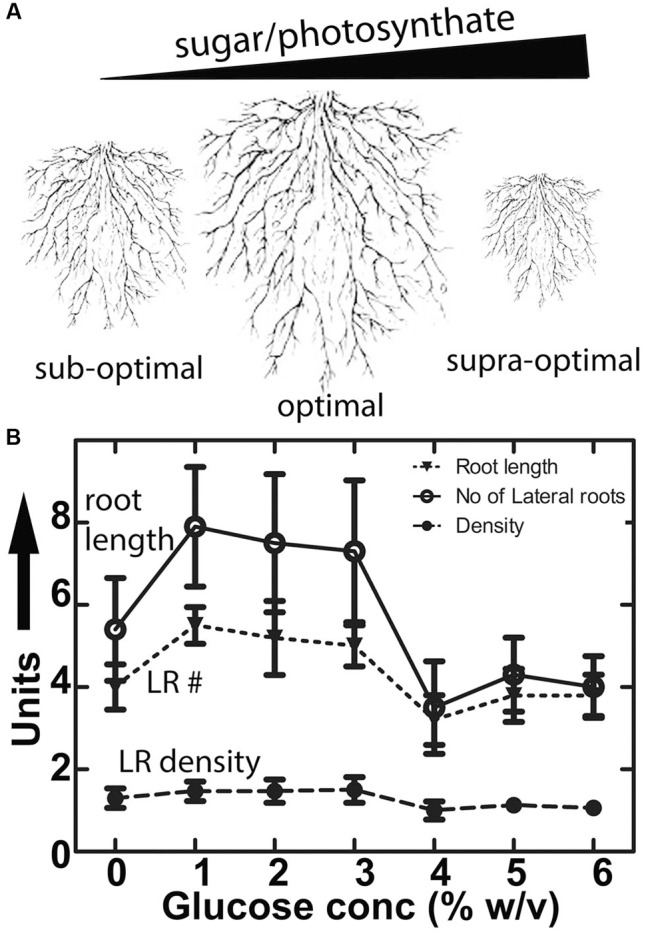
**Conserved architecture of plant root growth in nature and the lab. (A)** Diagrammatic representation of roots of different size showing constant lateral root density and lateral root primordial position grown under sub-optimal, optimal and supra-optimal nutrient conditions. **(B)** Primary root length (mm), number of lateral roots (per root) and lateral root density (roots per mm) in Col-0 in the presence of different concentrations of glucose. Values represent the mean of 3 independent experiments (*n* = 10 each); bars represent the standard error.

An intrinsic characteristic of any plant species is its root system architecture (RSA). Although RSA is plastic in development, the features that constitute the RSA, such as lateral root density and lateral root primordial position, remain constant over different root mass volumes (**Figures 1A,B**) (Dubrovsky et al., 2006; Moreno-Risueno et al., 2010; Dubrovsky et al., 2011; Szymanowska-Pulka, 2013). Environmental variables for RSA are light, water, and nutrients. Given that roots are subterranean, the light effect is most likely due to the amount of sugar in the form of fixed carbon (photosynthate) that roots receive (Kircher and Schopfer, 2012; Colaneri and Jones, 2014; Gupta et al., 2015). Sucrose is produced in the cytosol of photosynthesizing cells and is the predominant sugar to be transported through phloem to the carbon sink tissues where this disaccharide sucrose is converted back to the monosaccharides, glucose and fructose, by cell wall invertases (Ruan, 2014).

Recently, an accumulating body of evidence suggests that sugars also function as signaling molecules on RSA (Hanson and Smeekens, 2009; Smeekens et al., 2010; Kircher and Schopfer, 2012; Moghaddam and den Ende, 2013). Under laboratory conditions, exogenous application of sugars (D-glucose and sucrose) at a low concentration stimulates primary root elongation and lateral root development (Freixes et al., 2002; Lee-Ho et al., 2007; Booker et al., 2010; Roycewicz and Malamy, 2012; Gupta et al., 2015).

An increase in the available photosynthate stimulates root development (Rogers et al., 2006; Hachiya et al., 2014). Specifically, elevated CO_2_ levels increase lateral root formation (Crookshanks et al., 1998; Smith et al., 2013). Reciprocally, nutrient deficiencies that increase the root-to-shoot ratio and alter RSA are associated with an accumulation of sugars (Liu et al., 2009; Giehl et al., 2014). While it is clear that photosynthetic rates in above-ground tissues are associated with the extent and pattern of growth in roots (Kircher and Schopfer, 2012), how at the cellular level this growth is coordinated remains unknown.

It was suggested that putative crosstalk between sugar and hormones, mainly auxin homeostasis/signaling triggers changes in RSA (Gupta et al., 2009, 2015; Mishra et al., 2009; Booker et al., 2010; Lilley et al., 2012; Sairanen et al., 2012). Auxin and sugar act in concert and the availability of free sugars regulate the biosynthesis and degradation of auxin (Lilley et al., 2012; Sairanen et al., 2012). The physiological role of this concerted auxin-sugar action is control of cell division and elongation (Wang and Ruan, 2013).

Plants have at least two glucose sensing pathways; one is metabolism based, mediated by HEXOKINASE1 (HXK1); (Cho et al., 2006, 2007) and the other is based on extracellular sugar mediated by the receptor-like protein called AtRGS1. In *Arabidopsis*, AtRGS1-mediated sugar sensing is coupled by the heterotrimeric G protein complex comprised of a Gα subunit (GPA1) and a Gβγ dimer (AGB1 and AGG, respectively) (Chen and Jones, 2004; Grigston et al., 2008; Phan et al., 2012; Urano et al., 2012a,b; Bradford et al., 2013). We previously established that loss-of-function alleles for AGB1 alter RSA by increased root mass and altered auxin signaling (Ullah et al., 2003). Synergism between auxin and glucose on root growth and lateral root formation is altered in *agb1* mutants indicating G protein action in RSA maintenance (Booker et al., 2010).

The present work provides data suggesting a G protein mediated signaling mechanism for photosynthate partitioning to roots. The heterotrimeric G protein mediates sensing of nutritional state/sugar levels that integrate sink carbohydrate levels to maintain root architecture. The G protein complex lies apically in the sugar pathway controlling photosynthate partitioning in lateral roots. More importantly, this study provides substantial support for G protein functioning as a sensor that integrates sink carbohydrate levels to maintain root growth, in which sugar acts as a signal to regulate transcriptional changes.

## Materials and Methods

### Accession Number Details of the Genes Used in the Study

HXK1, At4G29130; RGS1, At3G26090; AGB1, At4G34460. All RNA-seq libraries produced in this study can be accessed at the NCBI Sequence Read Archive under accession number SRP059460 or at the link http://www.ncbi.nlm.nih.gov/sra/?term=SRP059460.

### Plant Material and Growth Conditions

*Arabidopsis thaliana* ecotype Columbia (Col-0) was used in this study unless otherwise indicated. The G protein mutants and transgenic lines were previously described (Ullah et al., 2003; Chen et al., 2006; Trusov et al., 2007). The *hxk1*-3 is a Columbia null allele (Huang et al., 2015). Seeds were germinated after stratification at 22°C under short-day conditions (8-h light/16-h dark, light intensity 200 μmol m^-2^s^-1^) or in the dark. The PIN2 reporter lines were described by Wisniewska et al. (2006). The *agb1*-2 null allele was introgressed by genetic crossing.

### ^11^CO_2_ Pulse Chase Experiment and Measurements of Sugars

The ^11^CO_2_ fixation experiment used 14 days-old Col-0 and *agb2-1* seedlings grown on MS plates under constant light. ^11^C, a short-lived radioisotope (*t*_1/2_= 20.4 min) was used to study the allocation and partitioning of [^11^C]-photosynthate. The high specific activity of ^11^C, allows a short 5–10 s pulse rather than a continuous stream of 1–2 h needed when using ^14^C. Given the high rate of transport of photosynthate observed for *Arabidopsis*, ^11^C provides greater temporal (for analyte) and spatial (for PET imaging) than ^14^C. ^11^C was made by irradiating a nitrogen gas (N_2_) target with 17-MeV protons from the TR-19 cyclotron (Ebco Industries) at Brookhaven National Laboratory to induce the ^14^N(*p*,α)^11^C nuclear transformation (Ferrieri and Wolf, 1983). Carbon dioxide labeled with ^11^C was captured on a molecular sieve (4 Å), desorbed, and quickly released into an air stream at 200 ml min^-1^ as a discrete pulse to the targeted seedling fixed inside a 5 × 10-cm airtight cell maintained at 21°C and fitted with red/blue light-emitting diodes (120 μmol m^-2^s^-1^) to ensure a steady level of fixation. In general, plants were pulsed with 20–30 mCi (740-1110 GBq) of ^11^CO_2_ as a 30-s pulse in a continuous stream of air. After pulsing, the seedlings were chased with normal air for 60 min. Roots and shoots were harvested at 20 and 60 min and placed into separate scintillation vials and radiation quantitated using a γ counter (Picker). For positron emission tomography imaging (PET), 3-week-old plants were transferred to the PET camera 10 min after pulsing. All radioactivity measurements were decay corrected to a standard zero time of each study to quantify allocation of ^11^C-photosynthate to the roots. After radioactivity measurements, ^11^C-labeled sugars and total sugars (^12^C) were analyzed by high performance thin layer chromatography followed by autoradiography as described by Babst et al. (2013). The sugar and the radioactivity data was normalized by the fresh weight of the tissue.

### Positron Electron Tomography (PET) Imaging

For PET imaging, a 30-s pulse of ^11^CO_2_ was administered to the youngest fully expanded leaf of a sorghum plant at grain filling stage. After 90 min incubation, the sorghum plant was scanned in a PET camera (HR+, SEMENS). The data was acquired over 30 min. The image was reconstructed and analyzed using an AMIDE medical image data examiner^1^. Validation of this method is described by Karve et al. (2015).

### Glucose Assays

To observe the effect of sugar on plant development, 4-day-old seedlings germinated on ½X MS media without sugar were transferred to plates containing various amount of sugars and grown vertically for 7 days. Primary root length and number of lateral roots were quantified using 10 seedlings per replicate and each experiment was repeated three times.

### Microscopy Imaging and Analysis

*Arabidopsis* PIN2-GFP in the Col-0 and *agb1*-2 backgrounds were imaged using a Zeiss LSM710 confocal laser scanning microscope equipped with an Apochromat40 (NA 1.2) water-immersion objective excited by a Multiline Argon laser (458/488/514 nm) excitation 488 nm and emission 520–560 nm. Fluorescence intensity measurements were performed with ImageJ (Albrechtova et al., 2014) and data was graphed with GraphPad Prizm (La Jolla, CA, USA).

### Auxin Analysis

Root tissue from the 7-day old seedlings was harvested below the root shoot junction, flash frozen in liquid nitrogen stored in 0.5 ml tubes in -80°C. Lyophilized samples were overnight shipped to the Department of Horticulture at the University of Minnesota where analysis for total and free auxin was performed exactly as described by Liu et al. (2012). The experiment was performed in triplicate. Each sample (treatment by genotype) had approximately 70–80 roots, roughly 25 mg of fresh weight.

### RNA Sample Preparation and Next-Generation Sequencing

Wild type and *agb1*-2 seedlings were grown vertically on ½ X MS, and 0.75% Phyto agar, 22° C, in the dark for 5 days followed by treatment with 2% glucose (gluc) in ½ X liquid MS for 4h in dark. The latter was achieved by pouring the liquid solution onto the plates which were then kept still for the 4 h duration. Control seedlings were treated with ½ X liquid MS. After treatment, the apical 1 mm region of roots, primarily the RAM, was harvested under a microscope using ultra sharp razor blades and snap-frozen in liquid nitrogen followed by RNA isolation using the RNeasy Plant Mini Kit (Qiagen) following the manufacturer’s protocol. Approximately 150 root tips were harvested per treatment by genotype.

mRNA-seq libraries were prepared with the Illumina TruSeq Stranded RNA library prep kit (RS-122-2201) as per the manufacturer’s protocol. One hundred nano-grams of total mRNA per sample were used in each preparation. Size selection (250–450 bp) was performed in each cDNA libraries using a 0.6X-0.8Xfd dual- Solid Phase Reversible Immobilization (SPRI) procedure provided by the manufacturer (SPRIselect reagent kit, item B23317, Beckman Coulter). A total of 12 libraries were prepared (two conditions, two genotypes, three replicates per genotype/condition) using different barcoded adaptors to allow the pooling of the libraries prior to sequencing. Quality control indicated that all libraries except one had >98% mapped sequence. The one library (*agb1*-2, control) that did not meet this condition was excluded, thus only two replicates were used for this condition.

### Gene Expression Analysis

The Illumina HiSeq2000 sequencer was used to generate an average of 55 million single-end reads (50 bp) for each of the libraries. The resulting RNA-seq reads were then aligned against the *Arabidopsis* genome (TAIR10) using TopHat (Trapnell et al., 2009). A maximum of two mismatches were allowed in the alignment and reads mapping to multiple positions in the reference were discarded. Reads mapping to each *Arabidopsis* gene were then counted by the HTSeq software (Anders et al., 2014) using default parameters. Differentially expressed genes between conditions were identified using the edgeR package (Robinson et al., 2010) with a false discovery rate (FDR) threshold of 0.05. A subset of 978 genes differentially expressed by the glucose treatment in at least one of the genotypes was submitted to hierarchical clustering based on the Euclidean distance of their *z*-score normalized expression values. Sets of genes belonging to sub-clusters in this analysis were submitted to Gene Ontology (GO – biological process) enrichment analyses using the PlantGSEA database (Yi et al., 2013) and the Bingo plugin for Cytoscape (Maere et al., 2005).

## Results and Discussion

### Sugar Control of Lateral Root Density Mediated by AGB1

**FIGURE 2:**
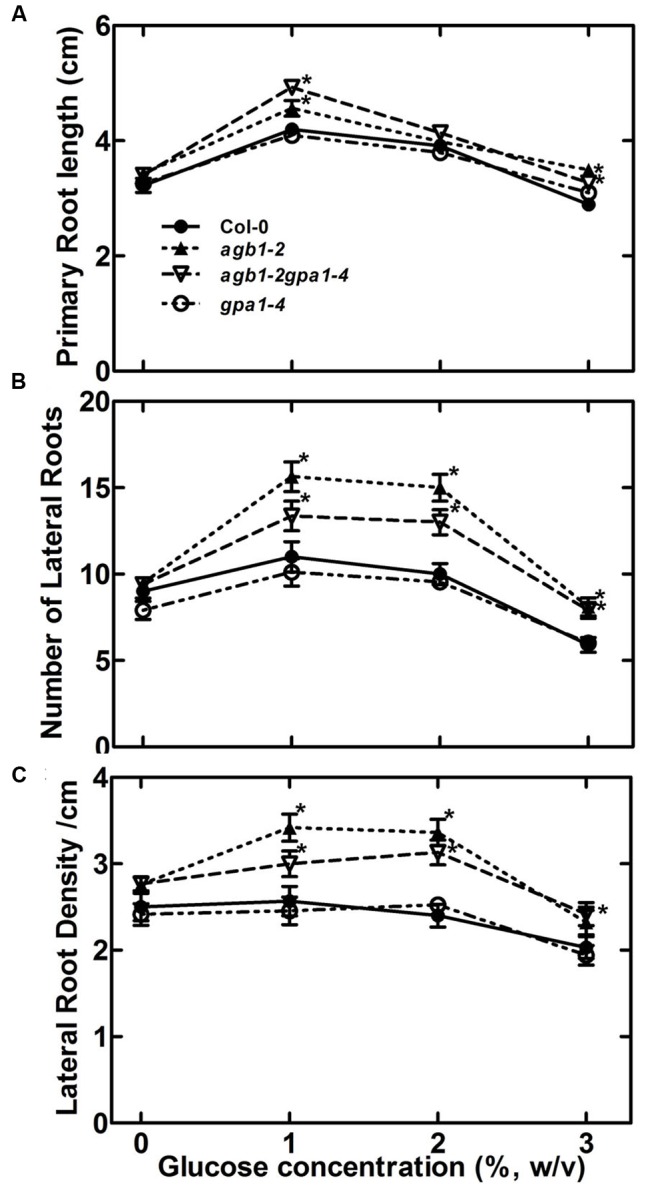
**Role of G protein subunits in sensing sugar in RSA maintenance. (A)** Primary root length of 11-day-old seedlings of Gα, Gβ and Gαβ double subunit mutants (indicated genotypes) were grown on ½ X MS, and 0.75% agar, 22°C, 8:16 light: dark cycle for 4 days followed by 7 days of vertical growth on different concentrations of glucose. **(B)** Lateral root number **(C)** Lateral root density. All experiments were repeated 3 times with 10–15 seedlings of each genotype per trial. Error bars represent standard error Student’s *t-*test results are based on difference between the wild type and indicated genotype shown as asterisks:^∗^*P* < 0.05.

Adding glucose or sucrose to media for optimal *Arabidopsis* seedling growth is standard lab practice but paradoxically it is not clear why 1–2% sugar in the agar medium is optimal since this amount does not occur in soils. The fact that there is an optimum concentration for root growth (**Figure 1A**) suggests that sugar is acting on RSA as a signal and not as a growth-limiting metabolite. **Figure 1B** shows that glucose both promoted and inhibited primary root growth and lateral root formation depending on the glucose concentration, but the overall architecture was not affected in wild type seedlings. That is primarily because while root length and lateral root number co-vary depending on glucose concentration, lateral root density remains constant for wild type (**Figure 1B**). We tested Gα (*gpa1-4*), Gβ (*agb1*-2) and Gαβ subunit double mutants (*agb1*-2*gpa1-4*) of the heterotrimeric G protein complex to discern any involvement of G protein subunits (**Figure 2**). We found that the developmental property of “fixed root density,” while developmentally plastic, is genetically encoded because loss of the Gβ caused lateral root density to increase with increased glucose amount (**Figure 2**, *P* = <0.005). To determine if this behavior is due to osmotic pressure, we tested root growth in the presence of various concentrations of the osmoticant mannitol and found that *agb1*-2 behaved like wild type (**Supplementary Figure S1**, *P* ≥ 0.1).

### Effects of Glucose on RSA of Sugar-Sensing Mutants

**FIGURE 3:**
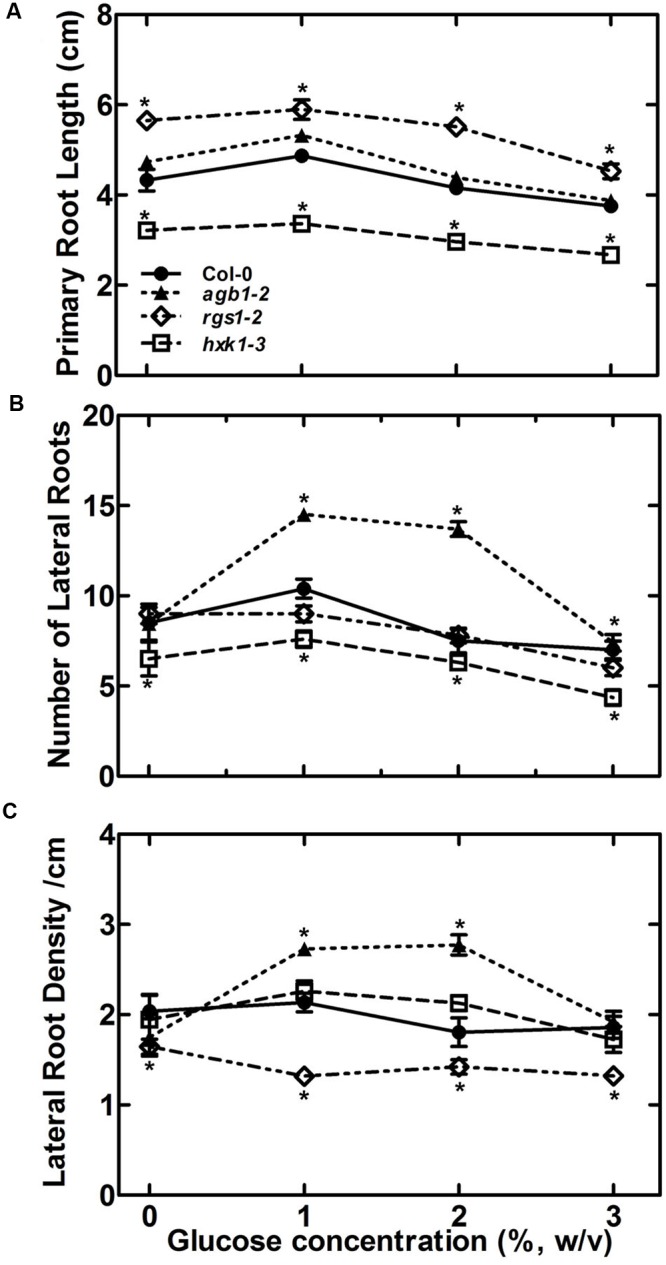
**The sugar sensing mechanism in RSA maintenance may involve both HEXOKINASE 1(*HXK1)* and REGULATOR of G SIGNALING 1 (*RGS1)*.** Effect of glucose on RSA in terms of primary root length **(A)**, number of lateral roots **(B)** and lateral root density **(C)** was compared for wild type, *agb1-2, hxk1-3* and *rgs1-2* mutant. All experiments were repeated three times with 10–15 seedlings used for each genotype in each trial. Error bars represent standard error Student’s *t-*test results are based on difference between the wild type and indicated genotype shown as asterisks: ^∗^*P* < 0.05.

Glucose modulation of the RSA (**Figure 1**) suggests the existence of a glucose-sensing mechanism that refines root development according to the amount of the translocated sucrose as the major form of assimilated carbon from source (leaves) to the sink tissue (roots). Phloem translocated sucrose is metabolized to glucose and fructose in the roots by invertases which determine sink strength. Both HXK1-dependent and -independent mechanisms contribute to glucose sensing in plants (Rolland et al., 2006; Hanson and Smeekens, 2009). Therefore, we performed phenotypic analysis on an *HXK1* null mutant (*hxk1-*3*)* and *AtRGS1* (*rgs1-*2*)*. Compared to its wild type Col-0, the *hxk1-*3 mutant displayed attenuated glucose effects, reduced primary root length and lateral root number (*P* ≤ 0.005) and showed insensitivity to glucose compared to the control (**Figure 3**). Root density of *hxk1*-3 was at the wild type level for all the tested glucose concentrations, however, this was because *hxk1* roots were not responsive to glucose with regard to both lateral root number and root length. Overall, the root system is poorly developed therefore it is difficult to conclude whether HXK1 plays a glucose signaling role or solely a metabolic role in roots (**Figures 3A–C**). Loss of *AtRGS1* conferred an increase in primary root length (*P* ≤ 0.005), insensitivity to exogenous glucose at the lower range (**Figure 3A**), and sugar-induced lateral root number compared to wild type (**Figure 3B**). Lateral root density of the *rgs1-*2 mutant was slightly lower over the entire tested range compared to *hxk1-*3 and Col-0 (*P* ≤ 0.005, **Figure 3C**). Therefore, we speculate that both HXK1 and RGS1 are involved in this glucose response but function differently.

### Dynamics of the Allocation of Leaf-Fixed Carbon to Roots

As discussed above, the evidence supports the conclusion that the source of sugars affecting RSA is from fixed carbon but it is unclear if the rate at which these sugars are produced is distributed to roots within a time scale over which G signaling operates (Fu et al., 2014). As discussed above, sugar strongly affected primary root growth and lateral root formation, but the overall RSA was not affected in wild type seedlings; i.e., the lateral root density was constant over a range of sugar concentration. Possible explanations are that AGB1 negatively regulates the amount of sugars fixed or affects the amount and/or gradients of auxin or both.

**FIGURE 4:**
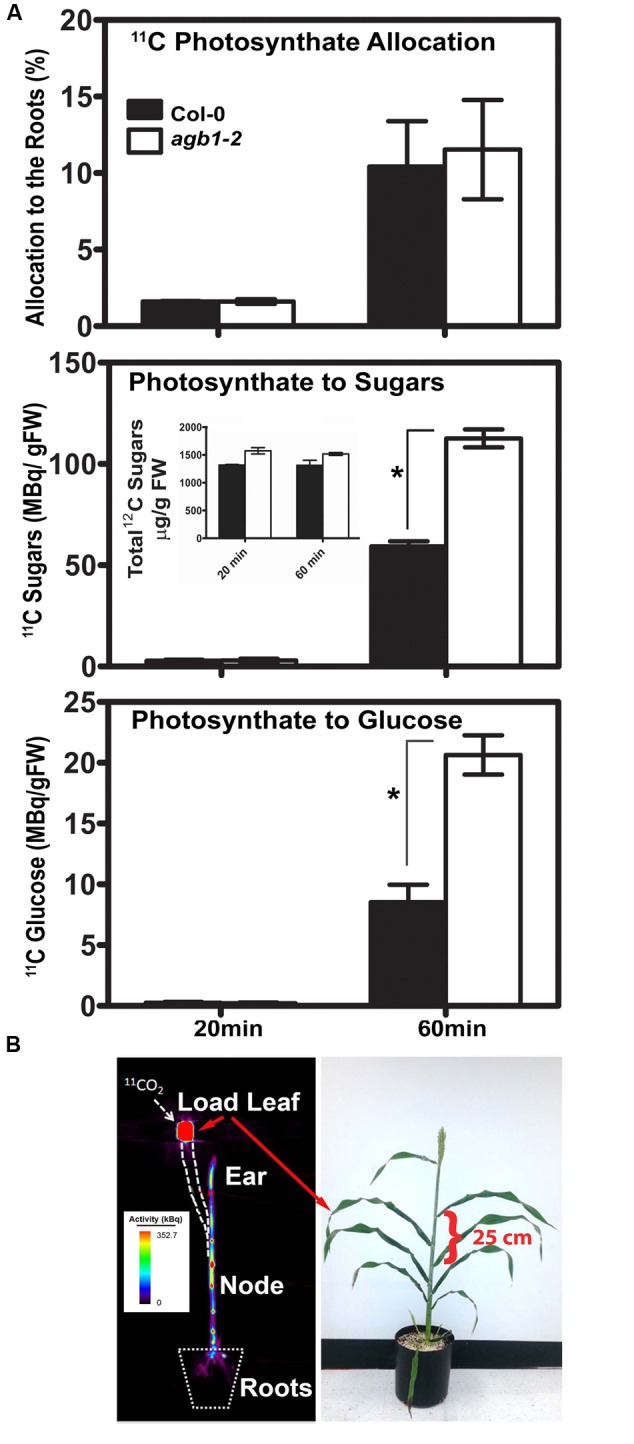
**Positron electron tomography imaging of allocation and partitioning of photoassimilate. (A)** Top panel. Total allocation of [^11^C] CO_2_ in the roots of Col (solid bars) and *agb1*-2 (open bars) at the indicated times. Middle panel. Partitioning of newly fixed ^11^CO_2_ to soluble sugars (glucose, fructose and sucrose) in the roots of Col (solid bars) and *agb1*-2 (open bars) at the indicated times. (Inset) Total non-radioactive [^12^C] sugars (glucose, fructose and sucrose) in the roots of Col (solid bars) and *agb1*-2 (open bars) at the indicated times. Non-radioactive [^12^C] sugars were extracted and analyzed by thin layer chromatography as described previously (Babst et al., 2013). Numbers represent the average of 3 independent experiments (*n* = 10 each) and error bars represent SE. Lower panel. Partitioning of newly fixed ^11^CO_2_ to glucose in the roots. Percentage values were calculated as radioactivity in the roots relative to the total seedling activity. Radioactivity (MBq/g FW) represent the mean of 3 independent experiments (*n* = 10 each) and error bars represent the standard error. **(B)** Fixed carbon is rapidly distributed to tissue sinks. (Left panel) The image shows the distribution of ^11^C-labeled photoassimilate in different parts of in an intact sorghum plant shown in the right panel imaged 2 h after ^11^CO_2_ administration to the load leaf at the position indicated. Heat scale represents activity/pixel. Load leaf = site of ^11^CO_2_ administration. Velocity was 1.25 cm min^-1^. A distance of 25 cm is indicated by the bracket. Student’s *t-*test results are based on difference between the means. Asterisks indicate *P* < 0.001.

To test the first possibility, we quantitated the photosynthesis-derived sugar flux to the roots in *agb1-2* and wild type Col-0 seedlings by γ counting (**Figure 4**). Roots may sense photosynthetic activity by the amount, duration and frequency of the sugar present. For this to operate, sugars from fixed CO_2_ must reach roots quickly enough (minutes) for root cells to be able to sense the dynamics of carbon fixation. Photosynthesis-derived sugar flux to the roots in *agb1-2* and wild type Col-0 seedlings was determined using 14-d-old seedlings treated with a pulse of the short-lived radiotracer ^11^CO_2_ and chased with ^12^CO_2_ (**Figure 4A**). Quantitation in harvested tissue was made by counting radioactive γ that are formed as a product of positron annihilation. After 20 min, approximately 2% of the radiolabeled photosynthate had already reached the roots in both Col-0 and *agb2-1* seedlings (**Figure 4A**, photoassimilate allocation). The rate of movement was estimated to be 0.5–0.8 cm min^-1^. By 60 min, the absolute amount increased approximately 5–6 fold, suggesting the time scale for a linear response is minutes.

The allocation of the newly fixed carbon to roots partitioned into at least three soluble sugars (glucose, fructose and sucrose). The total amount of [^11^C] photosynthate partitioned to the roots did not differ between the two genotypes at the tested time points, indicating comparable photoassimilate allocation in the absence of AGB1 (**Figure 4A**). However, after 60 min, almost twice as much [^11^C] glucose, [^11^C] fructose, and [^11^C] sucrose in the *agb1-2* roots was found compared to Col-0 (**Figure 4A**, photosynthate to sugars = 3 hexoses combined, *P* < 0.001). However, total sugars (i.e., not immediately fixed ^11^C) were not statistically different between genotypes (**Figure 4A**, photosynthate to sugars inset) indicating that over time the difference in the fixed sugars reached a new equilibrium. Most of the difference in the fixed [^11^C] sugars was due to an increased amount of [^11^C] glucose (**Figure 4A**, photosynthate to glucose, *P* < 0.001).

Attempts to visualize allocated carbon in *Arabidopsis* by positron emission tomography (PET) did not yield sufficient resolution due to the small plant size. Irrespective of the size of the plant, phloem sap flow velocity varies only slightly between diverse plant species (Windt et al., 2006). Therefore, to visualize and quantitate this rapid carbon allocation, we used sorghum because of its larger size, in particular the greater distance between the leaf and sink tissues. The first fully expanded mature leaf of sorghum during a grain filling stage was pulsed with ^11^CO_2_ for 30 sec. PET images were taken 90 min after ^11^CO_2_ exposure. High levels of radiation were detected in the stem, particularly high at nodes, and in the grain head. Remarkably ^11^CO_2_ was observed ∼100 cm away from the loading site traveling at a rate of at least 1.25 cm min^-1^. Photoassimilate was observed in the roots within minutes (**Figure 4B**).

### Feedback Loop Controlling Glucose Economy

There are three possible explanations why the *agb1* mutant root had higher levels of sugar: (**1)** less sugar is secreted from the *agb1-*2 root compared to the wild type. Root exudation plays a major role in maintaining root-soil contact by modifying biochemical and physical properties of the rhizosphere. Compounds such as amino acids and, to a much lesser degree, sugars are secreted by plants roots in order to promote microbial and fungal growth (Chaparro et al., 2014). (**2)** It is possible that the flux of glucose, fructose and sucrose to the roots is higher in the *agb1*-2 mutant. If this were true, we would expect to see a higher total of fixed ^11^CO_2_ in the root yet this did not occur (**Figure 4A**). (**3)** It is possible that sugar metabolism is altered in the *agb1*-2 mutant. We favor this last which is consistent with our observation that 22% of the interacting partners to the G protein core described in the G protein interactome are annotated as “metabolic processes” (Klopﬄeisch et al., 2011). This includes half of the enzymes in glycolysis, two enzymes in the Krebs cycle, and one cytosolic enzyme in the glucose shunt (Colaneri and Jones, 2014). Our previous study using *promoter-AGB1*::GUS lines showed that AGB1 transcript levels are sugar inducible in the root tip indicating involvement of feedback loops (Mudgil et al., 2009).

### Sugar Effect on Auxin Levels in AGB1 Mutant Roots

There exists a complex interplay between glucose and auxin in the regulation of root (Mishra et al., 2009; Booker et al., 2010) and shoot development (Mishra et al., 2009; Booker et al., 2010; Barbier et al., 2015). In addition, both sugar and auxin increase lateral root number (Boerjan et al., 1995; Mishra et al., 2009; Kircher and Schopfer, 2012; Roycewicz and Malamy, 2012). Glucose induces the expression of a subset of genes involved in auxin biosynthesis pathways, and auxin biosynthesis and metabolism rates corresponds to endogenous hexose levels (Sairanen et al., 2012).

**FIGURE 5:**
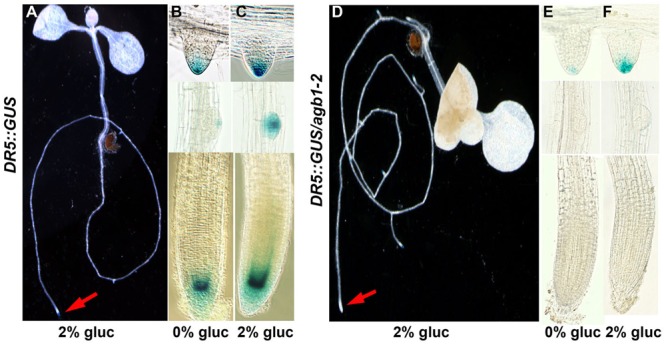
**Glucose-induced auxin maxima in *agb1*-2 mutant roots.** *DR5::GUS* is a synthetic, auxin-inducible gene promoter reporter used to detect auxin maxima or auxin signaling. **(A–C)** Wild type and *agb1*-2 **(D–F)** seedlings were treated with 2% glucose (gluc) for 4 h **(A,C,D,F)** and compared to the untreated controls **(B,E)**. Arrows point to the root tip. Compared to wild type, glucose did not increase DR5-driven expression of GUS in the *agb1*-2 root tips (*cf*. **B,C**; lower panels to **E,F**; lower panels) and lateral root primordial (*cf.* **B,C**; middle panels to **E,F**; middle panels), although *DR5::GUS* expression occurred in emergent lateral roots (*cf.* **B,C;** upper panels to **E,F**; upper panels).

The G protein complex may mediate a sugar-induced increase in auxin level by increased auxin synthesis overall and/or increased auxin maxima patterning by altering auxin transport. Genetic ablation of *AGB1* confers enhanced basipetal auxin transport (Mudgil et al., 2009) and sugar increases basipetal auxin transport associated with increased auxin levels (Mishra et al., 2009; Sairanen et al., 2012). Moreover, local auxin gradients generated by directional transport of auxin coincide with the site of organogenesis (Benková et al., 2003; Dubrovsky et al., 2008). Based on these findings, we hypothesized that AGB1 plays an important role in setting up local auxin gradients in response to glucose and therefore modulate expression of auxin-responsive genes. To test this hypothesis, we used the auxin reporter *DR5::GUS* that measures overall transcriptional output of auxin signaling (Friml et al., 2003; Sauer and Friml, 2011). To examine the effect of sugar on local auxin gradients/signaling, 5-day-old wild type and *agb1*-2 seedlings were treated with D-glucose (2%) for 4 h in the dark. In wild type roots, glucose stimulates *DR5::GUS* staining in different regions of the root (RAM, initiating primordia and emerged roots) (**Figures 5A–C**), whereas in the absence of AGB1, *DR5::GUS* expression was undetectable in the RAM (cf. **Figures 5A,D**, red arrows), and absent in the early stages of lateral root primordia development whether or not treated with sugar (cf. **Figures 5B,C** with **Figures 5E,F** center panels), whereas the RAM of the emergent lateral roots showed glucose-induced *DR5::GUS* expression (**Figures 5E,F**, top panels). These results indicate that AGB1 is necessary to attain sugar-induced, local auxin maxima/signaling at the site of cell division in the primary RAM and in the early stages of lateral root initiation. The absence of DR5::GUS staining in seedlings lacking AGB1 indicates either reduced auxin at the meristem or attenuated auxin signaling.

To distinguish between these two possibilities, we measured total endogenous auxin level in 5-day-old roots (**Supplementary Figure S2**). No significant difference in auxin level was found between *agb1*-2 and wild type roots or between control and glucose-treated roots (*P* > 0.05). This indicates that total auxin *per se* is not important for altering RSA and that auxin distribution and/or auxin signaling is glucose and AGB1 dependent.

### AGB1 Mediated Glucose Sensing Converge on PIN2 Protein Localization

**FIGURE 6:**
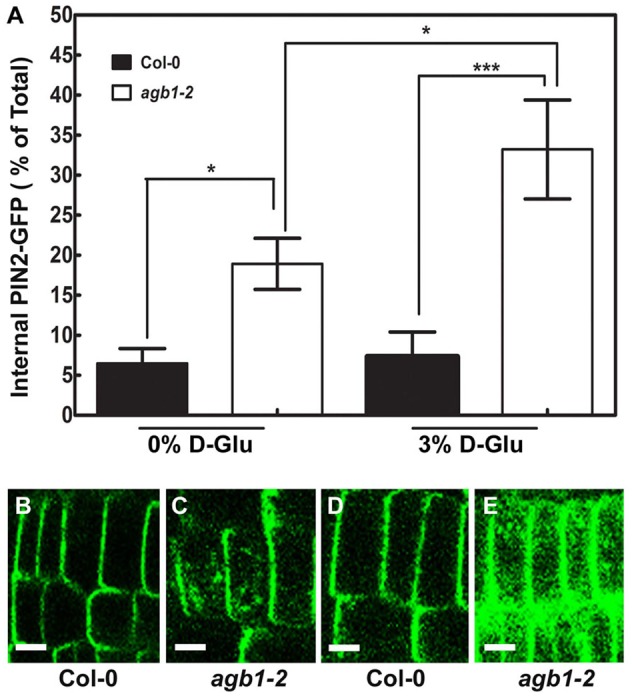
**Altered subcellular localization of PIN2-GFP in the *agb1*-2 mutant. (A)** Quantitation of PIN2-GFP subcellular localization in Col-0 and *agb1-*2 root tip cells grown with or without supplemental 3% glucose. Fluorescence intensities in multiple seedlings were measured using ImageJ software and compared using Student’s *t-*test, shown as asterisks:^∗^*P* < 0.05; ^∗∗∗^*P* < 0.0005. PIN2-GFP localization in Col-0 root tip cells **(B,D)** grown without (-) or with (+) supplemental 3% glucose, respectively. **(C,E)** Amount of PIN2-GFP internalized in *agb1-*2 root tip cells grown without (-) or with (+) supplemental 3% glucose, respectively. Scale bars represent 5 μm. In order to compare directly these genotypes, *PIN2-GFP*/*agb1-*2 lines were obtained by crossing *agb1-2* plants into the *PIN2-GFP* line shown in panels B and D. This experiment was repeated three times and reproducible *PIN2-GFP* localization pattern was observed upon glucose treatment.

The polarity of the auxin transport facilitator PIN2 is regulated by auxin, ethylene, cytokinin, strigolactone, and light (Habets and Offringa, 2014; Koltai, 2014; Luschnig and Vert, 2014). These signals regulates PIN action in the root (Wisniewska et al., 2006; Ruzicka et al., 2007; Laxmi et al., 2008; Ruzicka et al., 2009; Ding et al., 2011; Shinohara et al., 2013). We previously reported that sugar signaling shows dose and duration reciprocity (Fu et al., 2014). We tested if changes in sugar levels affect polar auxin transport via G proteins by controlling PIN2-GFP protein level/localization expressed under the control of the native PIN2 promoter (**Figure 6**). Glucose (3%) caused little or no detectable change in PIN2-GFP localization (*P* > 0.05) in the wild type root (*cf.* **Figures 6B,D**, quantitation in **Figure 6A**). In the *agb1-*2 roots, the internal PIN2-GFP level was higher compared to Col-0 (*cf.* **Figures 6B,C**), consistent with our previously reported increase in basipetal auxin transport in *agb1*-2 (Mudgil et al., 2009). In contrast to the lack of an effect in wild type, the 3% glucose treatment in the *agb1-*2 mutant further increased the amount of internalized PIN2-GFP which appeared as a punctate pattern in these cells (*P* < 0.05) (*cf.* **Figures 6C,E**) indicating AGB1 attenuates sugar-mediated PIN2-GFP localization/ recycling.

### Sugar-Mediated Auxin Signaling in the AGB1 Mutant

**FIGURE 7:**
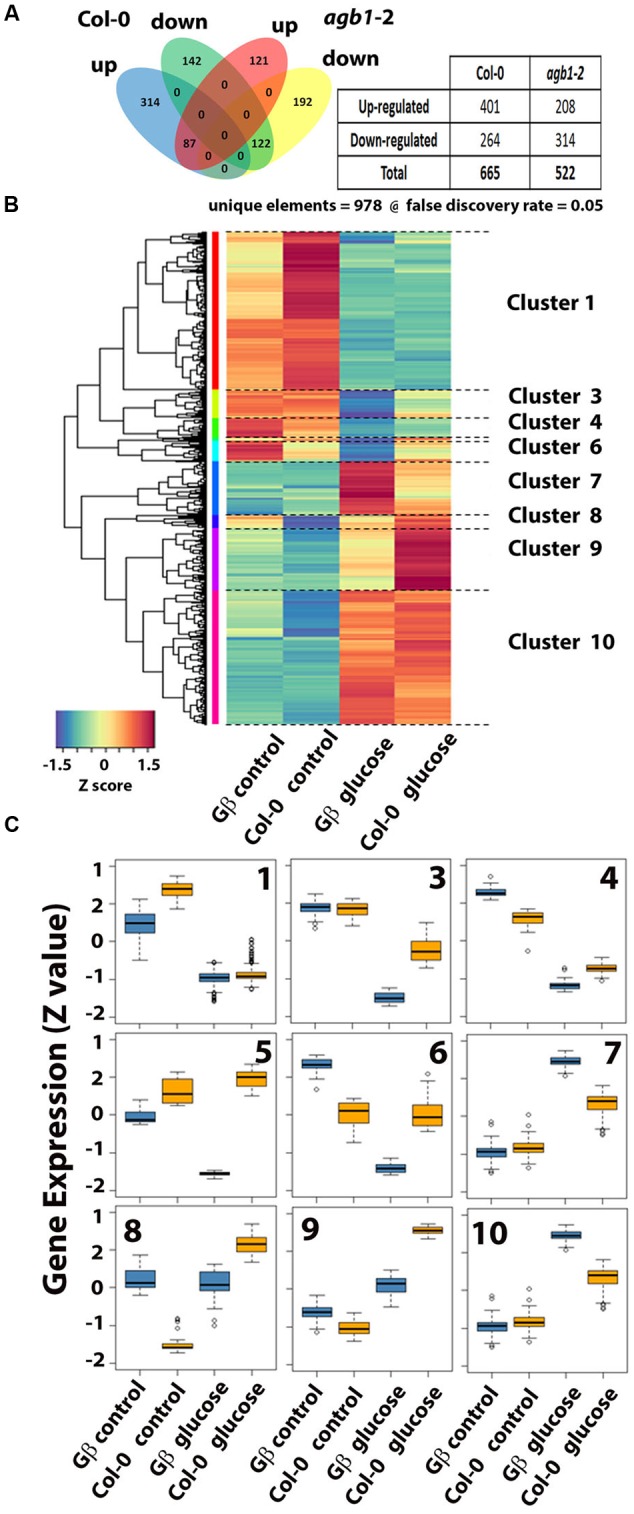
**Glucose-induced gene expression in the 1-mm root tips of wild type and *agb1*-2 mutant.** Five-day old, etiolated seedlings were treated with glucose for 4 h and the apical 1-mm of roots was harvested for RNA profiling as described in Materials and Methods. **(A)** Venn diagram quantitating genes that were differentially expressed in the two genotypes. The table inset summarizes the number of genes scored as up or down regulated in each genotype. **(B)** Cluster analysis of the genes that have altered expression displayed as a heat map. Ten distinct clusters were formed. Clusters 2 and 5 had too few genes to label. **(C)** The expression profile of each cluster is shown as a box plot. The top of the rectangle indicates the third quartile, the horizontal line indicates the median, and the bottom of the rectangle indicates the first quartile. The vertical line from the top indicates the maximum value, and the other vertical line extending from the bottom indicates the minimum value.

We previously showed that a set of auxin-regulated genes are misregulated in the *agb1*-2 mutant, including genes known to be important for lateral root development (Ullah et al., 2003). Therefore, RNA-Seq was used to test the hypothesis that AGB1 mediates glucose regulation of gene expression in the 1-mm apical root tip. Triplicate libraries of each control and glucose treated roots were prepared, bar-coded and subjected to sequencing resulting in at least 40 million and as high as 65 million reads for each library. Such high coverage enabled us to have confidence in low expressed genes while maintaining a stringent FDR setting of 5% (FDR = 0.05). There were 978 unique elements (**Figure 7A**). Glucose-repressed genes were similar in the two genotypes. There were 264 genes repressed by glucose in Col-0 and 314 in *agb1*-2. The genotypes shared 122 of these genes and the remaining are exclusive of each genotype (i.e., 142 in Col-0 and 192 in *agb1*-2). A greater difference between genotypes was observed for glucose-induced genes. There were 401 genes that increased expression upon glucose treatment in wild type. There were 208 up-regulated by glucose in *agb1*-2 with only 87 of them shared by Col-0 and the remaining 121 were statistically significant in *agb1*-2 only. This analysis indicated that AGB1 plays a direct or indirect role in glucose-regulated gene expression.

Gene Ontology analysis revealed that genes repressed by glucose independently of G signaling are related to metabolism of several types, including sucrose, organic acids, and amino acids (**Supplementary Table S1**). Genes with increased expression by glucose independently of G signaling are primarily related to cell wall processes. Genes for which AGB1 is required for proper expression are related to responses to stress. Genes with expression that was repressed compared to wild type were predominantly related to stress responsiveness. The GO analysis indicated that AGB1 is positively regulating genes that are involved in biotic stress, both innate immunity and effector-induced defense (**Supplementary Table S1**, underlined annotations, 23 of 37 genes with *P* < 0.003). This is consistent with the hyper susceptibility of the *agb1* mutants to *Pseudomonas*, necrotrophic and hemibiotrophic fungi, and oomycetes (Urano et al., 2013). AGB1 negatively regulates genes involved in photon capture (**Supplementary Table S1**, underlined annotations, 8 of 24 genes with *P* < 0.002) and genes related to biotic stress (10 of the remaining 16, *P* < 0.002). While it is known that *agb1* mutants have altered sensitivity to several abiotic stresses (Urano et al., 2013), a role for AGB1 in photosynthesis has not previously been reported. AGB1 control of photosynthesis genes seems relevant but it is surprising for these genes to be altered in the root tip.

Annotations provide a generalized view of pathway function; therefore we used “genotype by treatment” cluster analysis of individual gene responses and presented it as a heat map (**Figure 7B**). We organized the 978 differentially expressed genes in our experiment into 10 clusters, with one cluster having only 2 genes (Cluster #2). The distribution of expression level for the individual cluster is compared across treatment and genotype. The PlantGSEA (Yi et al., 2013) server returned informative function information for the gene cluster confirming the analysis performed on the 978 unique elements (**Figure 7A**). Clusters 4 and 6 share overlap with gene sets involved in plant defense, cluster #1 overlaps with genes involved in sugar signaling and metabolism, cluster #3 with plant hormone pathways, cluster #7 with protein folding, and cluster #10 with light and ROS responses.

The hypothesis is that glucose acts on auxin signaling output through apical auxin signaling, therefore the Aux/IAA gene family was inspected closely for differences in glucose regulation between Col-0 and the *agb1*-2 mutant (**Supplementary Figure S3**). Among the *IAA* genes, some were regulated by glucose and all of these in the *agb1* mutant were comparable in expression to wild type. While *IAA4, IAA5, IAA6* appear to be different from wild type, the differences were not supported statistically. Although one possible difference between genotypes may be with *IAA34*. In the control condition, *IAA34* was ∼3 fold repressed in *agb1*-2 relative to Col-0. While the FDR is not significant (FDR = 1), the p-value is 0.054991, supporting a possible trend, albeit weak. *IAA34* is one of two *IAA* genes encoding IAA proteins lacking the destruction box, DII. No functional information on IAA34 is available at this time to enable speculation on whether or not it could be a component of glucose-induced, AGB1-affected lateral root formation. On the other hand, *IAA19*, which is known to play a role in lateral root development and shown here to be glucose induced does not require AGB1. Therefore, we conclude that glucose induction of the IAA gene family members is not a prominent part of the AGB1 mechanism.

Because members of the recently described central regulator PHYTOCHROME-INTERACTING FACTOR (PIF) sense changes in sugar levels and regulate the RSA (Salisbury et al., 2007; Kircher and Schopfer, 2012; Lilley et al., 2012), we examined the expression of the 7 PIF genes but found these all to be poorly expressed in root tips (RPKM < 1.0) and there were no differences between wild type and *agb1*-2.

**FIGURE 8:**
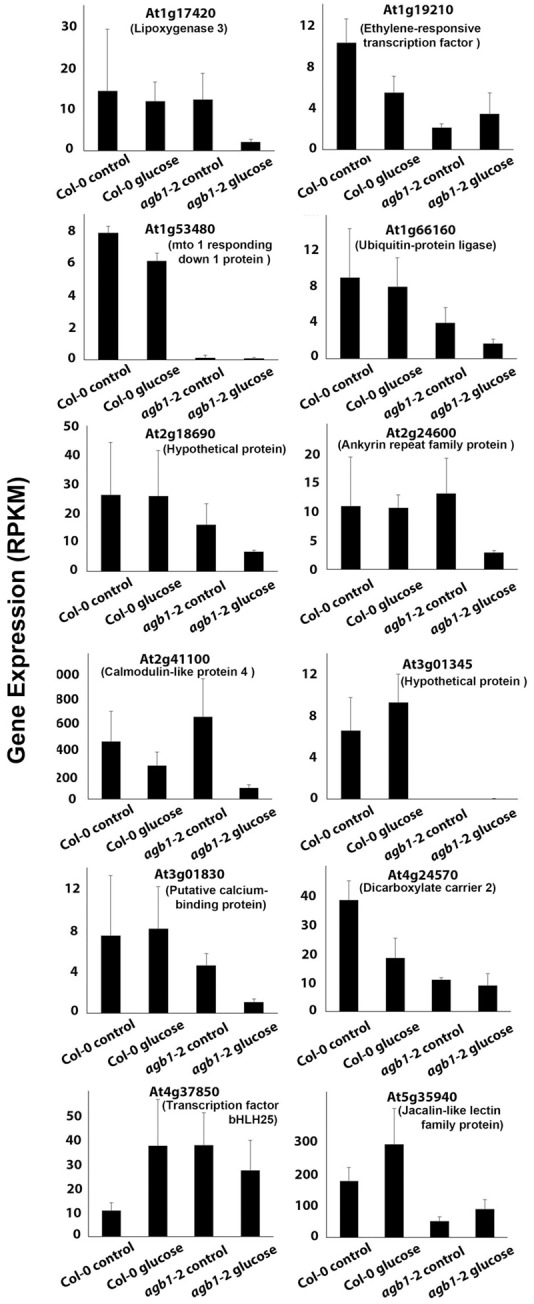
**Glucose regulation of genes that differ between wild type and *agb1*-2 root tips.** Genes that are differentially expressed (FDR ≤ 0.05) between the two genotypes in at least one of the conditions (control and glucose treatment) are shown. Normalized gene expression is shown as reads per kb per million reads (RPKM).

At least 12 well-expressed genes with profound difference in glucose responsiveness in the *agb1*-2 mutant relative to wild type were identified (**Figure 8**). Many other genes were noted but did not meet the threshold of expression (RPKM >1.0). Two genes that were completely repressed in the *agb1*-2 mutant are at loci At1g53480 and At3g01345 (**Figure 8**). At3g01345 encodes a putative *O*-glycosyl hydrolase. This is interesting because AGB1 is required for the expression of the *0*-glycosyl transferase, *TBL26* (Grigston et al., 2008), although the significance of glycosyl modification in AGB1-modulated lateral root formation is not presently obvious. At1g53480 encodes MRD1 (*MTO*1 responding down). This gene is down-regulated in the *mto1*-1 mutant that over-accumulates soluble methionine. AGB1 interacts with ARD1 in the methionine salvage pathway in *Arabidopsis* (Friedman et al., 2011), but again, the significance of this connection is not obvious. Among the other profoundly misregulated genes (**Figure 8**), At1g66160 physically interacts with AGB1 (Kobayashi et al., 2012). This gene encodes a U-box ligase. Both AGB1 and this E3 ligase play roles in innate immunity (González-Lamothe et al., 2006; Gilroy et al., 2011; Yaeno et al., 2011; Liu et al., 2013; Lorek et al., 2013; Torres et al., 2013), suggesting convergence between root development and defense.

### Summary

While it is accepted that the root system is a sink for photosynthesis-fixed sugars (Freixes et al., 2002; Macgregor et al., 2008), it was not known if these sugars act as signaling elements to control the architecture of the root. We showed that RSA is genetically encoded and that one of these genes points to signaling mediated by the heterotrimeric G protein pathway. We established that G proteins sense the dose of the sugar signal/carbon nutrient status in roots (which operates under dose and duration constraints) and positively affect RSA (**Figures 1** and **2**).

We found higher levels of glucose, fructose, and sucrose in the absence of the G protein beta subunit, the *agb1* mutant root. It is possible that sugar metabolism is altered in the *agb1*-2 mutant. Considering the velocity at which sugars arrive from the mesophyll to the root system (**Figure 4**), it is plausible that roots monitor photosynthesis with a resolution of minutes to hours. This is certainly the case for the shoot although monitoring occurs with a much longer time scale (Mason et al., 2014). Mason and coworkers showed that sugars are directly responsible for release of axillary bud dormancy. Because AGB1 acts like a negative regulator of lateral root density, G proteins may dampen the fluctuation of sugars and in that sense is part of a fluctuation sensor.

We established that G proteins mediate the glucose effect on RSA (**Figure 2**), through auxin patterning and transcriptional control (**Figure 6**, **7**, and **8**). Both HXK1 and RGS1 are involved in this glucose response but function differently (**Figure 3**). AGB1 plays an important role as a sensor component of glucose or carbon nutrient status in roots and modulates root growth. The steady-state levels of soluble sugar are higher in the absence of AGB1, indicating positive regulation within this pathway. Because glucose affects the posttranslational stability of N-MYC DOWN REGULATED LIKE 1 (NDL1) protein (Mudgil et al., 2009, 2013) in an AGB1-dependent manner, NDL1 stability may be part of this proposed feedback mechanism.

This work raises the possibility that fixed sugars derived through photosynthesis act as signals that regulate the RSA and we speculate that the roots may also signal back. This feedback from roots to shoots already has been proposed in a different signaling pathway in which AGB1 is also involved. Applied methyl jasmonate (MeJA) increased the allocation of ^11^C-labeled photosynthate to sink leaves and roots (Ferrieri et al., 2013). Because chilling the roots to 5°C inhibited the MeJA-induced increase allocation to sink leaves, feedback signaling from the roots was proposed. While AGB1 was not shown to be directly involved in this particular MeJA pathway, it was shown that AGB1 is essential for MeJA signaling in fungal resistance (Llorente et al., 2005; Trusov et al., 2007, 2009).

## Author Contributions

YM designed and conducted experiments, analyzed data, wrote and edited the manuscript (final root assays, prepared root tips for cDNA libraries and auxin quantification, PIN, DR-5-GUS localization); AK edited the manuscript and conducted experiments (PET assay, γ quantitation, sugar analyses); PT edited the manuscript and performed all the bioinformatics analyses; KJ performed experiments (preliminary root growth assay); MT-O analyzed data (PIN2 DATA analysis); AJ designed and performed experiments, analyzed data, wrote the manuscript, and managed the project.

## Conflict of Interest Statement

The authors declare that the research was conducted in the absence of any commercial or financial relationships that could be construed as a potential conflict of interest.

## Footnotes

1 http://amide.sourceforge.net/

## Supplementary Material

FIGURE S1 **G protein sensing glucose levels is specific and not shown by osmotic control mannitol (A)** Primary root length **(B)** Lateral root number **(C)** Lateral root density of the various G protein mutants genotypes used in **Figure 2**. Glucose was replaced with the corresponding concentrations of mannitol. Experiments were performed three times with10–15 seedlings used for each genotype. MIXED ANOVA analysis followed by box cox test (Box and Cox, 1964) indicated that there was no significant difference between genotypes on different concentrations of mannitol (*P* > 0.1).

FIGURE S2 **Auxin measurements in the whole root of *agb1-2* and Col-0 in the presence and absence of 2% D-glucose.** Physical quantitation was performed by mass spectrometry as described in the Materials and Methods. Total free IAA is displayed as ng per g fresh weight of root tissue in whisker plots. Horizontal lines = means, bars represent STD. Symbols are the individual measurements. ANOVA analysis indicated that all values are not statistically different (*P* < 0.05).

FIGURE S3 ***IAA* genes expression in wild type and *agb1-2* mutant without (control) or with glucose treatment in 1/2 MS liquid without sugar.** Expression profile of 28 out of 29 analyzed *IAA* genes showed no difference in expression upon glucose treatment in *agb1* mutant background compared to wild type. Minor visible differences were not supported statistically. Only one *IAA, IAA34* showed ∼3 fold repression in *agb1*-2 compared to Col-0 upon glucose treatment. While the FDR is not significant (FDR = 1), the *P*-value is 0.054991.

TABLE S1 **Gene Ontology (GO) Analysis Genotype by treatment**.
