# Supplementary figures and images for: Photosynthate Regulation of the Root System Architecture Mediated by the Heterotrimeric G Protein Complex in Arabidopsis
Source: Front Plant Sci. 2016 Aug 25;7:1255. doi: 10.3389/fpls.2016.01255 (PMC4997095; doi:10.3389/fpls.2016.01255)

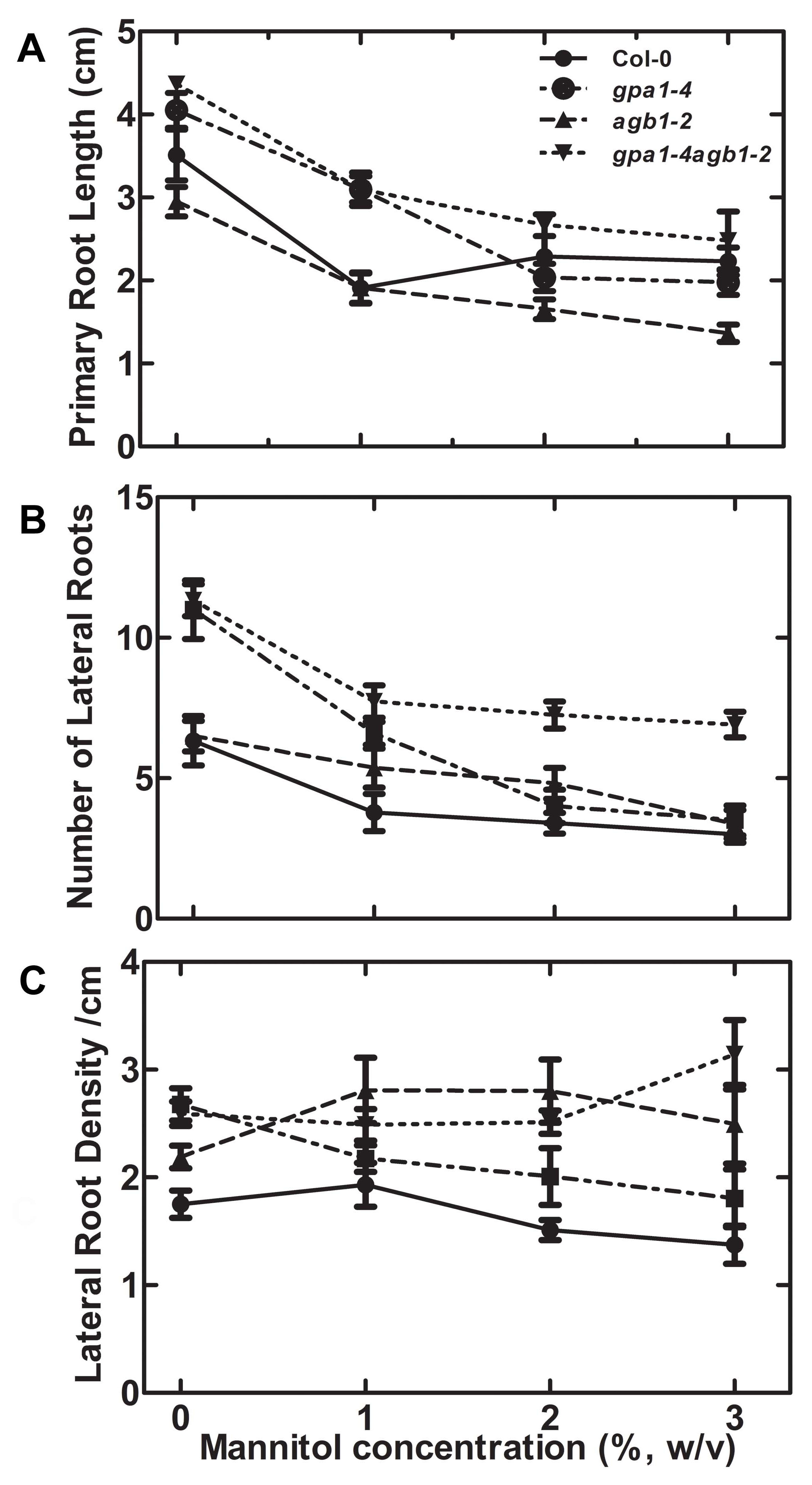

Supplement: FIGURE S1 — G protein sensing glucose levels is specific and not shown by osmotic control mannitol (A) Primary root length (B) Lateral root number (C) Lateral root density of the various G protein mutants genotypes used in Figure 2. Glucose was replaced with the corresponding concentrations of mannitol. Experiments were performed three times with10–15 seedlings used for each genotype. MIXED ANOVA analysis followed by box cox test (Box and Cox, 1964) indicated that there was no significant difference between genotypes on different concentrations of mannitol (P > 0.1). [file Image_1.JPEG]

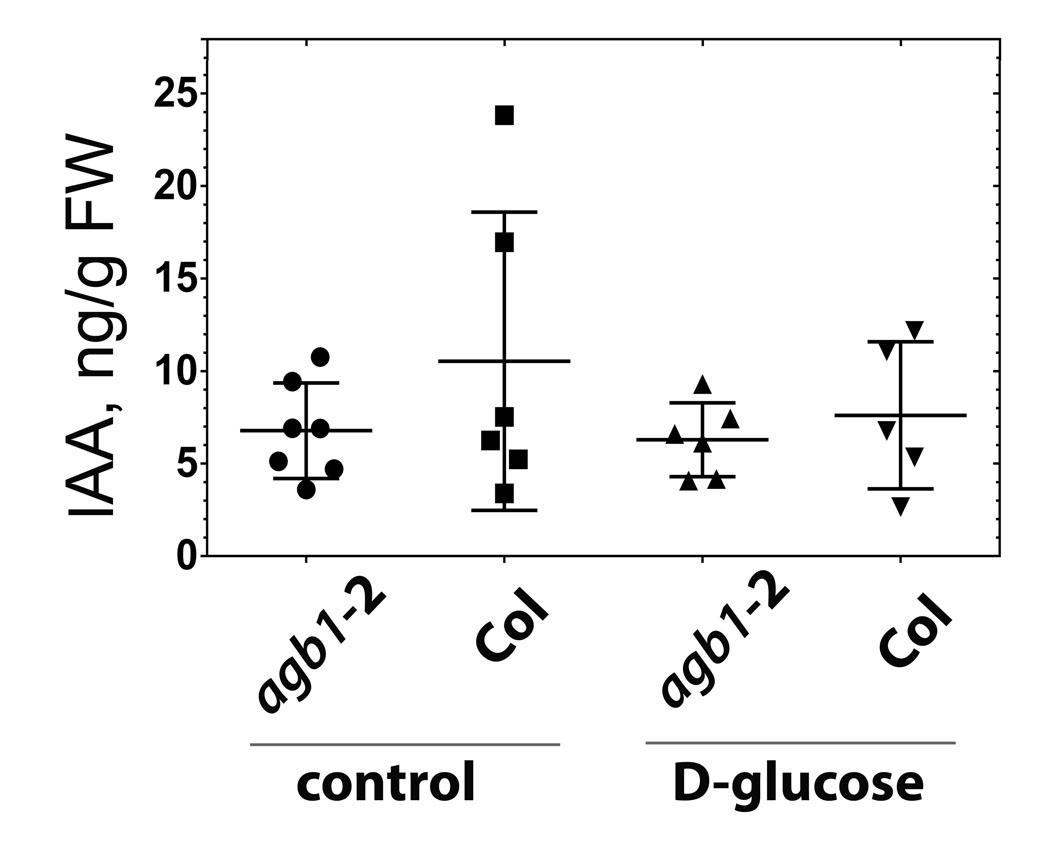

Supplement: FIGURE S2 — Auxin measurements in the whole root of agb1-2 and Col-0 in the presence and absence of 2% D-glucose. Physical quantitation was performed by mass spectrometry as described in the Materials and Methods. Total free IAA is displayed as ng per g fresh weight of root tissue in whisker plots. Horizontal lines = means, bars represent STD. Symbols are the individual measurements. ANOVA analysis indicated that all values are not statistically different (P < 0.05). [file Image_2.JPEG]

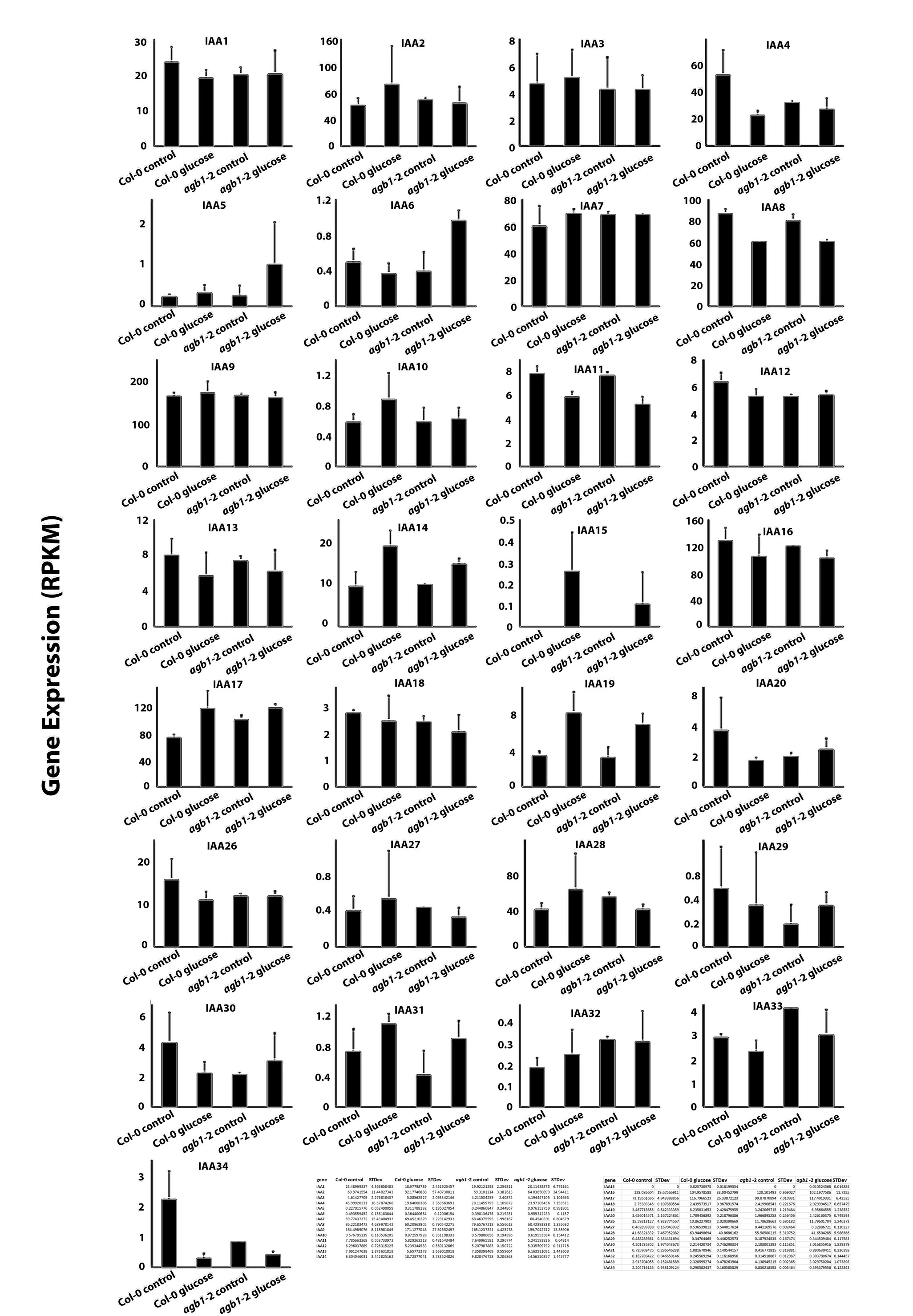

Supplement: FIGURE S3 — IAA genes expression in wild type and agb1-2 mutant without (control) or with glucose treatment in 1/2 MS liquid without sugar. Expression profile of 28 out of 29 analyzed IAA genes showed no difference in expression upon glucose treatment in agb1 mutant background compared to wild type. Minor visible differences were not supported statistically. Only one IAA, IAA34 showed ∼3 fold repression in agb1-2 compared to Col-0 upon glucose treatment. While the FDR is not significant (FDR = 1), the P-value is 0.054991. [file Image_3.JPEG]
